# Multiple Chronic Wounds in an Immunocompetent Patient With Severe Major Depressive Disorder: A Case Report

**DOI:** 10.7759/cureus.77667

**Published:** 2025-01-19

**Authors:** Emmanuel Tito, Rekha Kalyani, Fatima Halilu, Lavet Tita, Ines Kafando

**Affiliations:** 1 Medicine, Johns Hopkins University School of Medicine, Baltimore, USA; 2 Psychiatry, Johns Hopkins University School of Medicine, Baltimore, USA; 3 Internal Medicine, Johns Hopkins University School of Medicine, Baltimore, USA; 4 Internal Medicine, Western Michigan University Homer Stryker M.D. School of Medicine, Kalamazoo, USA

**Keywords:** chronic non healing wounds, delayed wound healing, depression, depressive patients, wounds and healing

## Abstract

Depression is a significant comorbidity among patients with chronic wounds. It is known to delay wound healing by interfering with adherence to medical interventions and impairing physical and cognitive functioning. This case report describes a 38-year-old immunocompetent woman who presented with multiple complex chronic wounds. The patient was admitted to the medical wards and subsequently diagnosed with severe major depressive disorder. Factors contributing to depression in individuals with chronic wounds are not well understood. We employed a multidisciplinary approach to wound care to address the factors that contributed to her presentation.

## Introduction

The Diagnostic and Statistical Manual of Mental Disorders, 5th Edition (DSM-5), defines depression as a mood disorder characterized by persistent feelings of sadness and loss of interest [1]. Major depressive disorder (MDD) is a severe form of depression characterized by persistent sadness, loss of interest, and other symptoms that significantly impair daily functioning [1-3]. These symptoms may include depressed mood, anhedonia (loss of interest or pleasure), changes in appetite or sleep, low energy, feelings of worthlessness, difficulty concentrating, and thoughts of death or suicide [1]. MDD typically lasts for at least two weeks [1].

Chronic wounds are defined as skin integrity loss with one or more underlying structures and absent healing within eight weeks [2]. They encompass foot ulcers, venous leg ulcers, and pressure injuries [3]. It is estimated that at least 30% of patients with chronic wounds suffer from depressive symptoms [4]. Depression is often associated with wound severity and is linked to reduced quality of life, increased morbidity, and mortality [4,5].

The association between chronic wounds and depression was first mentioned by House and Hughes in 1996 [5], and has since been investigated by other studies [6]. While the association between depression and delayed wound healing is recognized, the clinical impact of severe, untreated depression on the trajectory of chronic wound management remains poorly understood [7]. This case report presents a 38-year-old woman with multiple complex chronic wounds who developed severe MDD. The objective of this report is to highlight the profound impact of untreated depression on the development and management of chronic wounds, emphasizing the importance of early identification and treatment of mental health conditions in this patient population. This case underscores the need for a multidisciplinary approach to care that addresses both the physical and psychological needs of individuals with chronic wounds.

## Case presentation

A 38-year-old Caucasian woman was brought to the Emergency Department (ED) at the request of her roommate for further evaluation of her worsening wounds. Her medical history included neurogenic bladder due to a previous cauda equina syndrome, resulting in wheelchair-bound status, depression, agoraphobia, chronic neuropathy, and C5-6 anterior discectomy and fusion due to cervical myelopathy. From the chart review, her prior cauda equina was managed non-operatively. There was no other relevant surgical history. Her substance use history was notable for tobacco use (approximately two to three cigarettes per day for 10 years) but negative for alcohol use. Family history was noncontributory. Current medications included escitalopram 10 mg daily and gabapentin 300 mg twice a day, but the patient was not adherent to her home medications. The patient was single and lived with a roommate. At baseline, her functional status was limited. She was able to transfer to and from her wheelchair independently and perform basic self-care activities such as washing her face and brushing her teeth. She also engaged in some light upper-body exercises while seated. She reported no contact with family members and was unable to provide details regarding her family history of psychiatric illness or her own prior history of depression, including severity and treatment modalities.

Although the patient could not recall the exact timeframe or events leading to the development of the wound, she attributed it to lying on her left side for five weeks. History was largely obtained from emergency medical services (EMS) due to the patient's limited participation in questioning. According to EMS, the patient lived with a roommate who provided much of her history. Per the roommate, the patient had stayed on the floor wrapped in blankets and had not gotten up for five weeks. She had been incontinent of stool and urine, leading to the worsening of old wounds on her right thigh and the development of new wounds on her left flank and thigh. Upon chart review, the patient had been previously hospitalized at a local hospital, four months prior to presentation. At that time, she was noted to have right thigh wounds that were debrided by plastic surgery. There was no mention of left-sided wounds.

In the ED, the patient had a maximum heart rate of 110 beats/minute, a blood pressure of 64/42 mmHg, a temperature of 34.1°C, and an oxygen saturation of 97% in ambient air. She was alert and awake but withdrawn during the interview. Examination findings were notable for multiple pressure wounds, including the right thigh, left ischium, right knee, left rib cage, left posterior thigh, left posterior shoulder, left buttock, left lower abdomen, right lower leg, and right heel. Wounds on the right thigh, left ischium, right knee, and left rib cage were all graded as stage IV pressure wounds with moderate amounts of serosanguineous drainage. The left posterior shoulder and right lower leg wounds were graded stage III with small serous drainage. The right heel wound was unstageable and the base of the ulcer was obscured by eschar, preventing accurate assessment of the wound depth. Cardiovascular, respiratory, and neurological exams were unremarkable.

At admission, laboratory studies (Table 1) revealed severe neutrophilic leukocytosis (white cell count: 45.9 × 10^3^/L, reference range: 4.0-11.0 × 10^3^/L), anemia (hemoglobin: 11.8 g/dL, reference range: 13.5-17.5 g/dL), thrombocytosis (761x10^3^/µL, reference range: 150-400 x10^3^/µL), decreased sodium level (132 mmol/L, reference range 136-145 mmol/L), and decreased total protein (4.3 g/dL, reference range: 6.4-8.2 g/dL). Lipase, potassium, and thyroid-stimulating hormone were within the reference range.

**Table 1:** Laboratory studies on admission.

Laboratory Tests	Patient Values	Reference Values
WBC Count (x10^3 ^/microL)	45.9	4.0-11.0
Hemoglobin (g/dL)	11.8	13.5-17.5
Platelet count (x10^3^/microL)	761	150-400
Sodium (mmol/L)	132	136-145
Total Protein (g/dL)	4.3	6.4 - 8.2
Lactic acid (mmol/L)	8.8	0.4-1.6
C-reactive Protein (mg/dL)	17.10	≤ 0.29
Erythrocyte Sedimentation Rate (mm/hr)	95	≤ 20
Creatine Kinase (u/L)	223	21-215
Venous pH	7.35	7.32-7.42
Bicarbonate (mEq/L)	14	21-32
Anion Gap	12	9-16

Additional studies revealed an elevated lactic acid (8.8 mmol/L, reference range: 0.4-1.6 mmol/L), elevated C-reactive protein (17.10 mg/dL, reference range: ≤0.29 mg/dL), elevated sedimentation rate (95 mm/hour, reference range ≤20 mm/hr), elevated creatine kinase (223 U/L, reference range: 21-215 U/L), venous pH of 7.35 (reference range: 7.32-7.42), bicarbonate of 14 mmol/L (reference range: 21-32 mmol/L), and a normal anion gap of 12 (reference range: 9-16 mmol/L). Influenza A/B, respiratory syncytial virus (RSV), and COVID-19 by polymerase chain reaction (PCR) were negative.

**Figure 1:**
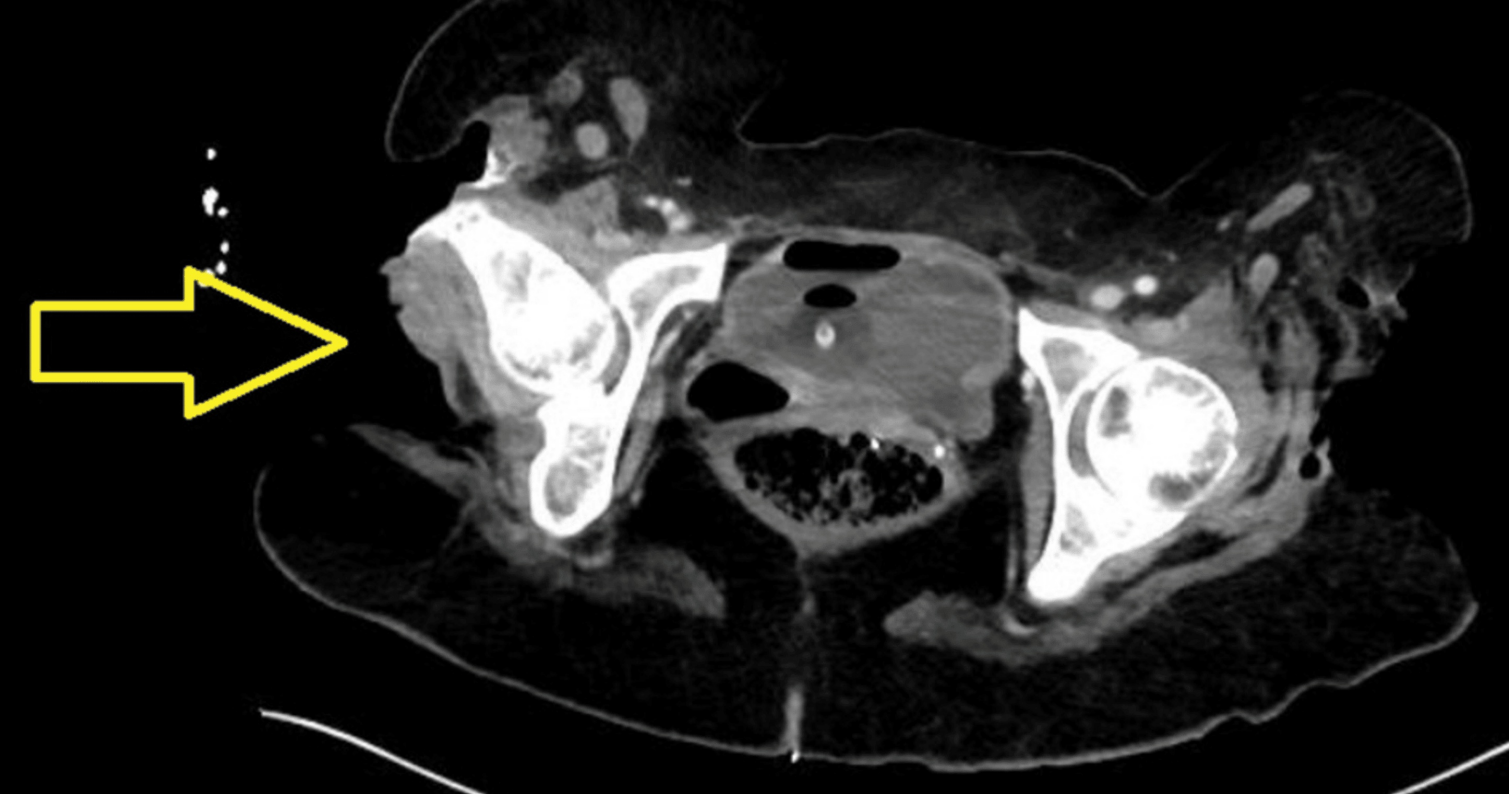
CT bilateral lower extremities with IV contrast revealing a large right wound of the right thigh extending from the iliac bone inferiorly to the right upper thigh approximately 21 cm in length (yellow arrow). There is air tracking superiorly along the gluteus musculature. There is exposed bone of the femur, with thinning and heterogeneity of the cortex of the exposed bone particularly at the greater trochanter. Minimal surrounding stranding of the soft tissues.

**Figure 2:**
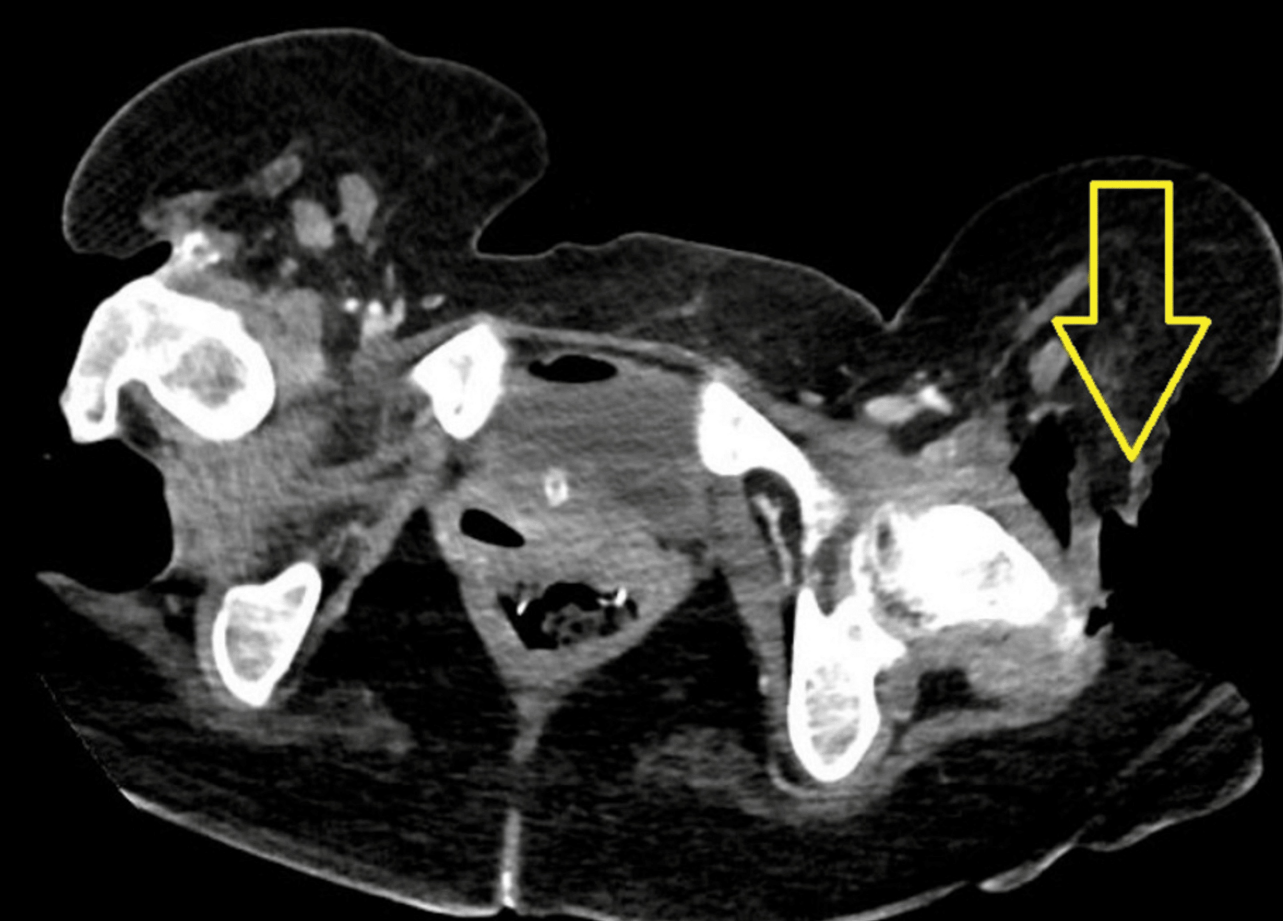
CT bilateral lower extremities with IV contrast revealing a large wound of the left thigh, subcutaneous emphysema tracking inferiorly along the gluteus musculature and quadriceps musculature (yellow arrow). There is exposed bone along the greater trochanter, with thinning and heterogeneity of the cortex of the exposed bone. Minimal surrounding stranding of the soft tissues.

A computed tomography (CT) scan of the chest, abdomen, pelvis, and lower extremities with intravenous contrast (Figures 1, 2) was remarkable for numerous wounds, including a left hip wound extending to the femur with emphysema along the quadriceps and gluteus musculature, a right hip wound with small-volume subcutaneous emphysema tracking along the gluteus musculature with exposed gluteal musculature and femoral bone, a large left lateral chest wall defect with exposed bone and subcutaneous emphysema tracking superiorly, and a right knee wound with exposure of the tibia.

The patient required decontamination prior to rooming due to extensive soiling of the skin and wounds with feces. Initial bedside debridement of the wounds on the patient's left lower extremity was performed by General Surgery in the ED. Despite receiving multiple boluses of lactated ringers (LR), the patient developed persistent hypotension. She was subsequently admitted to the medical intensive care unit (MICU) where she received a total of 3 liters of LR and initiated on norepinephrine infusion at a starting dose of 4 mcg/min for 22 hours.

Based on the patient's initial presentation, the differential diagnosis included necrotizing fasciitis in the setting of catatonia. Other potential explanations for the patient's symptoms at the time of presentation included cauda equina syndrome, spinal cord infarct, and myelopathy, all of which were less likely given the clinical history, physical exam, and imaging results. The patient's Bush-Francis Catatonia Rating Scale (BFCRS) score was 10, indicating potential catatonia. This score was derived from 2 points for immobility/stupor, 3 points for mutism, 3 points for withdrawal, and 2 points for autonomic abnormalities. However, a formal psychiatric evaluation did not meet the diagnostic criteria for catatonia as outlined in the DSM-5 [1]. The patient did not exhibit rigidity or waxy flexibility, which are more specific features of catatonia. Initially, her presentation was attributed to hypoactive delirium secondary to septic shock, metabolic acidosis, and multiple wounds. Her home medicine, escitalopram 20 mg, was restarted once daily, and the patient was scheduled for operative room (OR) debridement by general surgery.

In the second week of hospitalization, a full psychiatric evaluation was performed given the patient's increased interaction. This evaluation included a thorough Mental Status Examination (MSE). The patient reported feelings of guilt, shame, low mood, tearfulness, anxiety, poor sleep, and poor appetite, all consistent with a depressive episode. The patient's limited participation in the interview and difficulty providing a detailed history suggested potential impairments in insight and judgment. Psychometric testing was not performed. Her psychiatric differential diagnosis included apathy related to possible previous traumatic brain injury (TBI), intellectual disability, major mood disorder, negative symptoms of psychosis (less likely), or substance use disorder that the patient was minimizing (which may have contributed to the wounds if xylazine or levamisole were present). TBI can have significant neurological and psychological consequences, including apathy (lack of motivation and interest). The patient's limited participation in the interview and potential difficulties with providing a detailed history could be attributed to cognitive difficulties resulting from a previous TBI. Though there was an initial concern of intellectual disability, improvement in her cognition was noted in the following interviews. While less likely, negative symptoms of psychosis, such as avolition (lack of motivation) and alogia (reduced speech), can present with similar features to apathy. However, the presence of other depressive symptoms makes this diagnosis less probable in this case. The patient was followed by the psychiatric team for an additional week for diagnostic clarification.

Her admission blood cultures grew *Pseudomonas aeruginosa* and *Alcaligenes faecalis*. A limited transthoracic echocardiogram (TTE) showed no vegetation. Right hip fluid, left hip tissue cultures, and other tissue cultures grew *P. aeruginosa*, methicillin-resistant *Staphylococcus aureus*, methicillin-susceptible *S. aureus*, *Citrobacter freundii*, *Enterobacter cloacae*, and *Escherichia coli*. She underwent incision and drainage (I&D) of the left hip, debridement of bilateral lower extremities, and left flank/chest wall down to the rib, with multiple OR cultures growing the same organisms as the blood cultures. The next day, she underwent a washout of bilateral thighs and the left flank. Wound vacuum-assisted closure (VAC) was placed on the left chest wall. A diagnosis of osteomyelitis and septic arthritis was established based on the positive bone and tissue cultures.

Due to concerns of progressive ascending weakness, magnetic resonance imaging (MRI) was ordered. An MRI of the complete spine with and without contrast showed significant atrophy of the spinal cord without signal abnormality. There was an abnormal signal involving the posterior paraspinal musculature in the neck and back with prominent muscular atrophy also appreciated with findings suggestive of myositis. Subsequent MRI myositis protocol was remarkable for diffusely increased T2 signal throughout the bilateral lower extremity musculature, consistent with myositis. Electromyography (EMG) revealed myopathic units in the upper extremities and no voluntary units with a preponderance of spontaneous activity in the lower extremity muscles. EMG findings were not consistent with motor neuron disease. Neurology suspected metabolic disease or mitochondrial cytopathies; as a result, a right upper extremity (RUE) biopsy was requested. Neurosurgery was consulted in this context. Neurosurgery performed a biopsy of the right biceps which showed severe type 2 fiber atrophy, a nonspecific finding that may be seen in severe deconditioning.

By week 6 of hospitalization, she developed worsening altered mental status (AMS) thought to be due to metabolic acidosis that required mechanical ventilation for 48 hours. AMS resolved after five days with medical management. The hospital course was further complicated by severe pharyngeal dysphagia with subsequent permanent percutaneous endoscopic gastrotomy (PEG) placement to meet nutritional goals. On week 8 of hospitalization, the patient underwent a pedicled flap of left latissimus and left thigh trochanteric reconstruction with vastus lateralis and fasciocutaneous flaps. Infectious disease recommended 6 weeks of IV vancomycin, cefepime, and metronidazole.

On Week 9 of hospitalization, she reported persistent feelings of guilt, shame, low mood, tearfulness, anxiety, poor sleep, and poor appetite, consistent with a depressive episode. Mirtazapine 7.5 mg nightly was initiated to help with her mood, anxiety, sleep, and appetite. She underwent physical and occupational therapy while in the hospital, gradually returning to her baseline activity. Outpatient cognitive behavior therapy was recommended. At the time of discharge, her depressive symptoms had significantly improved with escitalopram and mirtazapine. Based on the psychiatric evaluation and clinical improvement, the final diagnosis was MDD. Throughout hospitalization, she was followed by multiple specialists including Plastic Surgery, General Surgery, Neurology, Neurosurgery, Psychiatry, Infectious disease, and Wound Care Nursing. After 84 days in the hospital, she was discharged to subacute rehab (SAR) for continued wound care and rehabilitation. Despite initial progress, she was unfortunately readmitted three weeks later due to worsening wound conditions. Due to transportation issues, the patient was unable to consistently attend outpatient follow-up appointments prior to readmission. After undergoing wound debridement, she was again discharged to SAR.

## Discussion

World Health Organization (WHO) states that depression is the leading cause of disability as measured by Years Lived with Disability (YLDs) and the fourth leading contributor to the global burden of disease [7]. This case report illustrates the substantial impact of untreated MDD on individuals with chronic physical conditions. Our patient, who had previously managed her chronic disabilities, experienced a rapid decline in physical health when depression set in. This led to pressure injury-related osteomyelitis, septic shock, and prolonged hospitalization. Our case uniquely highlights a direct causal link between depression and physical deterioration, where prolonged immobility induced by depression resulted in pressure wound development [8]. While depression more commonly delays wound healing or increases recurrence, our patient's case underscores the severity of untreated depression.

The bidirectional relationship between MDD and chronic medical conditions is well-established. Physical illnesses can exacerbate depressive symptoms, while untreated depression can worsen disease outcomes [7]. MDD often leads to adverse health behaviors, such as poor treatment adherence, reduced physical activity, and suboptimal self-care [9], all of which were evident in our patient's case. The intricate mechanisms underlying the interaction between depression and chronic diseases involve genetic, psychological, and neurobiological factors. Genetic predispositions and adverse life events can increase vulnerability to depression, which, in turn, can lead to unhealthy lifestyle choices and exacerbate chronic conditions. Neurobiologically, depression can dysregulate the hypothalamic-pituitary-adrenal axis, elevate proinflammatory cytokines, and impair autonomic nervous system function, thereby hindering wound healing [10,11].

Individuals with chronic illnesses are two to three times more likely to be diagnosed with MDD compared to their age- and gender-matched peers [12]. Despite this increased prevalence, MDD often goes unrecognized and undertreated in patients with chronic diseases. Diagnostic challenges arise when the physical manifestations of chronic illness mask depressive symptoms [12,13]. In our patient's case, decreased mobility and self-care were initially attributed to her physical conditions, delaying the recognition of depression's role in her deterioration. A multidisciplinary approach is crucial for managing patients with comorbid MDD and chronic diseases. Integrating mental healthcare with medical management can improve treatment adherence, reduce hospitalizations, and prevent relapses. In our patient's case, timely psychiatric intervention, coupled with aggressive wound care and rehabilitation, led to improvements in both physical and mental health.

Addressing the psychological aspects of chronic disease is essential for optimal outcomes. Effective physician-patient communication and early recognition of psychological distress can improve treatment adherence and enhance recovery [14]. This case underscores the need for healthcare providers to remain vigilant for signs of depression in patients with chronic physical conditions and to implement comprehensive care strategies that address both physical and mental health.

## Conclusions

Despite significant advancements in wound care and our understanding of chronic wound pathophysiology, complex wounds remain a substantial challenge for healthcare providers. A critical factor often overlooked is the interplay between chronic wounds and mental health, particularly depression. Research indicates that wound duration can contribute to the development of depressive symptoms. Conversely, existing depression can exacerbate wound healing and hinder self-management efforts. A multidisciplinary approach is essential for addressing both chronic wounds and depression in patients. Early screening for depression, followed by appropriate treatment, can enhance adherence to wound care regimens, improve physical and cognitive functioning, and ultimately reduce morbidity and mortality associated with chronic wounds.
